# Prevalence of musculoskeletal conditions and related disabilities in Bangladeshi adults: a cross-sectional national survey

**DOI:** 10.1186/s41927-020-00169-w

**Published:** 2020-12-16

**Authors:** Ahmad Zahid-Al-Quadir, M. Mostafa Zaman, Shamim Ahmed, Mahfuzur Rahman Bhuiyan, Md Mujibur Rahman, Ismail Patwary, Bidhu Bhushan Das, Shaikh Amir Hossain, Sujat Paul, Abu Shahin, Moshiur Rahman, Syed Atiqul Haq

**Affiliations:** 1 Department of Rheumatology, Bangabandhu Sheikh Mujib Medical University, Dhaka, Bangladesh; 2 WHO Bangladesh, 10 Gulshan Avenue, Road Number 5, Gulshan 1, Dhaka, 1212 Bangladesh; 3 Department of Medicine, Dhaka Medical College, Dhaka, Bangladesh; 4 Department of Medicine, Sylhet Women’s Medical College, Sylhet, Bangladesh; 5 Department of Medicine, Rangpur Medical College, Rangpur, Bangladesh; 6 Department of Medicine, Khulna Medical College, Khulna, Bangladesh; 7 Department of Medicine, Chittagong Medical College, Chittagong, Bangladesh; 8 Department of Medicine, Rajshahi Medical College, Rajshahi, Bangladesh; 9 Department of Medicine, Patuakhali Sadar Hospital, Patuakhali, Bangladesh

**Keywords:** MSK conditions, Disability, Prevalence, Bangladesh

## Abstract

**Background:**

Nationally representative data on burden of musculoskeletal conditions (MSK) in Bangladesh are not available. The objective of this study was to determine the prevalence of MSK conditions and related disabilities in the adult population of Bangladesh.

**Methods:**

A total of 2000 individuals aged 18 years or older were targeted from 20 primary sampling units (urban and rural) of all former seven divisions of Bangladesh in 2015. Structured interviews were done using the modified Community Oriented Program for Control of Rheumatic Disorders questionnaire to detect positive respondents. Standard criteria were used for diagnosing MSK conditions by rheumatology residents. In case of uncertainty, opinion was taken from senior rheumatologists. A *Bangla* version of the Health Assessment Questionnaire was used to determine disability.

**Results:**

A total of 1843 (92.1%) participated. Among them, 892 men and 951 women participated from rural (*n* = 716) and urban (*n* = 1127) areas. Their mean age was 40.5 (standard deviation 14.7) years. Almost a third did not have any formal schooling. Overall, 30.4% (95% confidence interval, 28.3–32.5) had MSK conditions. Low back pain (18.6%, 16.9–20.5), knee osteoarthritis (7.3%, 6.1–8.5) and soft tissue rheumatism 3.8% (2.9–4.7) were the three top-ranking MSK conditions. Rheumatoid arthritis (1.6%, 1.0–2.1), spondyloarthritis (1.2%, 7–1.8) and adhesive capsulitis (1.4%, 0.9–1.9) were relatively uncommon. Among those who had MSK conditions, 24.8% (21.3–28.6) had some degree of disability. Of them, 24.4% (21.0–28.1) had history of work loss during last 12 months.

**Conclusions:**

The high burden of MSK conditions and related disabilities in Bangladesh warrants greater attention of the health system. Further studies are needed to estimate the impact of this group of conditions particularly addressing related disabilities and loss of work.

## Highlights

1. This is the first national level study on musculoskeletal conditions using primary sampling units of Bangladesh Bureau of Statistics engaging rheumatology residents for data collection. The diagnoses have been validated or confirmed by rheumatologists in the field in partnership with divisional level medical college hospitals.
2. Prevalence of musculoskeletal conditions (low back pain, knee osteoarthritis and soft tissue rheumatism are top three conditions) is high. Their contribution to the disability and work loss makes them important to warrant public health and clinical interventions.

## Background

Musculoskeletal conditions are the most common cause of severe long-term pain, physical disability and early deaths [1, 2]. They affect hundreds of millions of people of all ages irrespective of social strata globally [3]. MSK conditions affect patients’ dexterity and functions, and thereby, impact their daily life activities. The most disabling conditions are back and neck pain, osteoarthritis, rheumatoid arthritis and fractures [2]. These conditions are often associated with major non-communicable co-morbidities (ischaemic heart disease, stroke, cancer and chronic respiratory disease) and they jointly augment disabilities and deaths [4, 5].

Pain is a common manifestation of musculoskeletal conditions. MSK is the commonest form of chronic pain for which people commonly seek medical help [6]. In addition, the MSK conditions impact quality of life [7]. These are, however, often not prioritized in the national policy making partly because these are costly and incurable [8]. Globally MSK conditions affect almost one in four adults with some regional variations. MSK conditions constitute the second cause of years lost due to disability (YLD) only after “mental and substance use disorders”. Disability adjusted life years (DALYs) due to MSK were ranked ninth among 23 major conditions categorized by the World Health Organization (WHO) in 2015 [9]. The prevalence of arthritis appears to be higher in the low- and middle-income countries (LMIC) compared to higher income countries [10]. Considering the significance of MSK conditions [11, 12], WHO launched the Community Oriented Programme for Control of Rheumatic Disorders (COPCORD) in 1981. After its launch many countries in the Asia Pacific [13–25] and Latin-America [26, 27] completed the COPCORD surveys. Many of the COPCORD studies revealed differences in prevalence between rural and urban areas and socio-demographic conditions [14–16, 18, 21, 23, 24].

Studies in two South-Asian countries, India and Pakistan, have reported a little lower prevalence [20, 21] than those reported from other regions. Institute of Health Matrix data indicate that low back pain was the topmost cause disability in 2007 in Bangladesh [28]. It continued to occupy the topmost position in 2017. Other MSK conditions ranked the 4th position in 2017 rising from the 5th position in 2007. One survey in Bangladesh conducted in two locations of Dhaka district reported a prevalence of 24% of MSK conditions [23]. Bangladesh is a country of 160 million people mostly living in rural areas but data from rural areas are not available. In addition, there is no nationally representative data on these conditions. MSK-related disabilities have never been studied at population level in Bangladesh. We have done this national level survey to determine the prevalence of MSK conditions and related disabilities in Bangladeshi adult population.

## Methods

This survey was designed to obtain national estimates on the burden of MSK conditions through a household level survey. Adults aged 18 years or more comprised the study population [29].

### Sample size and sampling

Assuming a point prevalence of MSK conditions among Bangladeshi adults of 24% [23], at 5% precision level, 280 participants were required in each reporting domain. Considering four reporting domains (rural-urban, male-female), a design effect of 1.5, and an 85% response rate, the calculated sample size for this survey was 1978. This was finally rounded to 2000.

The primary sampling units (PSUs) in Bangladesh constitute the sampling frame of national or subnational surveys. We used the PSUs of 2001 Census stratified in to the then seven divisions and rural and urban areas [30]. *Mauza* and *Mahalla* in rural and urban areas, respectively, were the PSUs with known boundaries. Maps with list of households of these PSUs were updated by the Bangladesh Bureau of Statistics. Total and urban rural population of the division were considered for allocating number of PSUs. Finally, 20 PSUs (8 urban and 12 rural) were selected and first consecutive100 households were included from each PSU. Households having even and odd numbers were assigned as male and female households to recruit one man and one woman, respectively, using the Kish table [31].

### Field team and its training

We employed seven field teams for seven divisions of Bangladesh. Each team consisted of one research physician (having at least one-year residency in rheumatology), one field organizer and two interviewers. The field team underwent a three-day training in Bangabandhu Sheikh Mujib Medical University before the pretest. All investigators and WHO technical team coordinated and conducted the training using a manual especially prepared for this survey. All investigators were present at the training sessions to ensure uniform understanding of procedures. After completion of the pretest, all investigators and the field had a one-day debriefing session for revising the manual and adjustment of the data collection tool. Another one-day refreshers training was done after completing one PSU by each team to minimize differences among teams.

### Survey instrument and data collection

The survey instrument was the modified COPCORD questionnaire [32]. The first part of the questionnaire aimed at detecting the respondents with musculoskeletal pain with some elaboration of the complaints. This portion was completed by the interviewers. The second part of the questionnaire had structured information for recording subjects’ history and clinical examination findings according to the COPCORD examination sheet. This was used by the research physicians for the diagnosis of conditions and detection of disability. The English version of the first part (that has been administered by the interviewers) of the questionnaire was translated to *Bangla*, then adapted and validated as per standard procedure [33].

### Field work

Data were collected in each PSU over a period of 6 days with engagement of the local community and health authority. The field organizer visited in advance and started household listing with the help of local health assistant on the first day. The field interviewers collected data (by reading out questions loudly to all participants), identified screening positive respondents, took physical measurements, and arranged interview with the research physician next 5 days. Two recall visits were done if the selected house was locked, selected person was not at home at the time of interviewer’s visit. They were declared non-respondents in case interview could not be done at the second recall. The research physician interviewed and examined the positive respondents for making a diagnosis. In doubtful cases, opinion of a division level investigator was taken. Investigators made at least one visit to PSUs in their respective divisions for validation of diagnosis. Erythrocyte sedimentation rate, C-reactive protein, rheumatoid factor and anti-citrullinated peptide antibody were tested in a pre-selected laboratory located nearby to aid the diagnosis. X-rays were also done as and when necessary.

## Operational definitions

### Covariates

The following variables were assessed as covariates for analysis: area of residence, sex, age, education, occupation, wealth index, body mass index (BMI). Education was categorized into four groups: no education, any primary education (completed grades 1–5), any secondary education (completed grades 6–10), and above secondary education (completed ≥ grade 11). Participants’ occupation was categorized into seven groups: home makers, laborers, business, salaried services, rickshaw/auto-rickshaw/van pullers, cultivators and others.

The wealth index was constructed using principal component analysis [29]. Asset information collected covered information on household ownership of 20 items, such as flush toilet, telephone, television, bicycle, sewing machine, bed. Each asset was assigned a weight (factor score) generated through principal components analysis. The scores were summed up for each household, individuals were ranked according to the total score of their households. The sample was then divided into four hierarchical groups from quartile one (lowest) to quartile four (highest).

Data on physical activity were collected based on self-report. First, respondents were asked the number of days they engaged in vigorous, moderate, or light physical activity throughout a typical week. Examples of vigorous, moderate and light activity were shown to the participants using showcards. Next, they were asked to estimate how many minutes per day they engaged in the activity. We then calculated metabolic equivalent tasks (MET)-minutes per week using the STEPwise Surveillance of noncommunicable disease risk factors (STEPS) protocol [29]. Finally, quintiles of MET-minutes were created, and the highest quintile was labelled as strenuous physical activity. Smoking habit was asked and recorded as current smoker, former smoker and non-smoker of any tobacco product such as cigarette, bidi and hukkah (water pipe).

History of physical trauma during last 12 months that needed medical treatment with or without residual damage, e.g., injuries due to accidents while travelling by road, trauma during occupational works while working in farming lands or factories, physical assault, etc., were obtained. Using height (meters) and weight (kilograms) measurements, we calculated BMI (weight/height^2^). People having BMI ≥25.0 were labelled as over-weight (this includes obese also). Random capillary blood glucose was measured. Diabetes was defined as blood glucose ≥11.1 or use of antidiabetic medication.

### Positive respondent

A subject was considered a positive respondent if he/she reported occurrence of pain at muscles, bones, joints, or any part of the body (musculoskeletal symptom) during the preceding 7 days. Subjects who did not report pain on those 7 days but were taking prescribed medicines for relieving pain, e.g., non-steroidal anti-inflammatory drugs or steroids, were also included. The respondents in whom musculoskeletal pain appeared, developed, or disappeared in the preceding 7 days were also labeled as a positive respondent.

### MSK conditions

All positive respondents were interviewed and thoroughly examined by the research physicians. Internationally accepted criteria [34–38] were used with adaptations whenever necessary. For conditions with no internationally accepted criteria and epidemiological definition, the clinical judgment of the research physician was used. In case uncertainty, opinion was taken from the investigators during their routine visit to respective PSUs. Following criteria were used for diagnosis of the MSK conditions:

1. Rheumatoid arthritis: 2010 American College of Rheumatology (ACR)/European League Against Rheumatism (EULAR) Classification Criteria [39];
2. Spondyloarthritis (axial and peripheral): Ankylosing Spondylitis Assessment Study (ASAS) criteria [40];
3. Ankysosing spondylitis: Modified New York Criteria 1984 [41];
4. Psoriatic arthritis: Classification Criteria for Psoriatic Arthritis (CASPAR) criteria [42];
5. Knee osteoarthritis: ACR clinical classification criteria for knee osteoarthritis (OA) [43];
6. Systemic Lupus Erythematosus: ACR Revised Criteria for the Classification of Systemic Lupus Erythematosus 1997 Systemic Lupus Erythematosus [44];
7. Soft tissue rheumatism: Commonly included subacromial bursitis, epicondylitis, trochanteric bursitis, anserine bursitis, and fibromyalgia [45];

Considering the limitations of investigations in the field situation, the differentiation between non-specific low back pain and lumbar spondylosis was not possible in many cases. Therefore, we have pooled these two together to report he prevalence. These are reported as low back pain.

### Disability and work loss

Disability was scored with a validated *Bangla* version of the Health Assessment Questionnaire (B-HAQ) [46]. This tool assesses the subjects’ level of functional ability and included questions of fine movements of the upper extremity, locomotor activities of the lower extremity, and activities that involve extremities. The B-HAQ included 20 items referring to basic activities of daily living, grouped into eight categories of functioning, viz., dressing and grooming, arising, eating, walking, hygiene, reach, grip and activities. Each category contained two or three specific component questions. Respondents are asked to rate the degree of difficulty they experienced in carrying out each activity on a 4-point rating scale: 0 (without any difficulty), 1 (with some difficulty), 2 (with much difficulty), and 3 (unable to do). The highest response in each category was divided by 8 to create a B-HAQ Disability index (B-HAQ-DI), yielding a total disability score of 0–3, where zero is no disability and 3 is severe disability [47]. Any one scoring ≥0.8 for B-HAQ-DI was categorized as having disability according to Quintana R et al. [48].

The recall period for determining work loss was 12 months. We have asked the participants whether they had to stop their usual occupational work, paid or unpaid (such as home makers), due to MSK conditions or related pain. Then the duration of such work loss (in days) was asked and recorded

## Statistical analysis

The data were entered into Excel spreadsheet and transferred to EpiInfo (version 7) for analysis. Missing values were identified to confirm the denominators, and consistency were checked.

All quantitative variables such as age, years of education, body mass index (BMI), B-HAQ-DI score were categorized before analysis. Alfa was set at 5% for considering statistical significance. Therefore 95% confidence intervals (CI) were calculated for all prevalence estimates such as MSK conditions, disabilities and related work loss. Results were presented for four reporting domains: rural and urban residential locations, and sex groups. Univariate logistic regression analysis was done for 11 candidate variables (age, sex, education, wealth quartiles, urban residence, smoking, strenuous physical activity, occupation, overweight, history of physical trauma, and diabetes) to get odds ratios (ORs) with their 95% CIs for MSK conditions combined (yes/no). Tri-variate logistic regression analysis was done for nine candidate variables (education, wealth quartiles, urban residence, smoking, strenuous physical activity, occupation, over-weight, history of physical trauma, and diabetes) to obtain age and sex adjusted odds ratios and their 95% confidence intervals of MSK conditions combined.

## Ethics approval and consent to participate

Ethical guidelines as outlined by the Declaration of Helsinki were followed throughout the study [49]. Ethical clearance was obtained from the Institutional Review Board of Bangabandhu Sheikh Mujib Medical University. Concurrence has been obtained from the local health authorities and elected representatives of the local government prior to data collection. Written (or thumb impression if unable to write) consent was obtained from the respondents in Bangla as per Institutional Review Board guidelines.

## Results

### Socio-demographic background

**Fig. 1:**
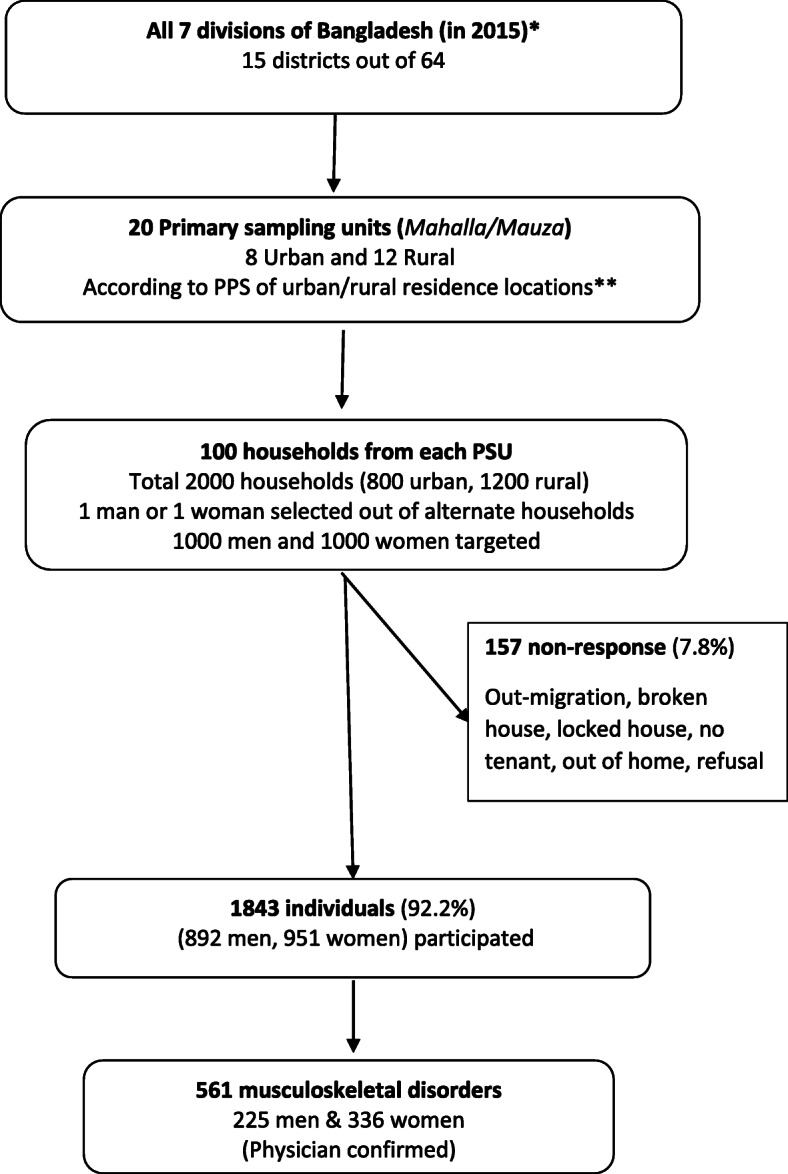
Flowchart for subject selection of the cross-sectional national survey on musculoskeletal conditions in Bangladesh, 2015. *Eight divisions from Sept 2015. PPS indicates population proportion to size. **Two recall visits were done if the selected house was locked, and selected person was not available at home at the time of interviewer’s visit. In case of non-particiaption after the second recall visit, the targeted household/individual was declared non-respondents

A total of 1843 respondents (aged 18 years or older) could be interviewed and examined out of targeted 2000 (response rate of 92.2%) as depicted in Fig. 1. The response rate was a little higher in the rural (93.9%) compared to the urban area (89.5%). There were 892 (48.4%) men and 951 (51.6%) women respondents. Mean age of the participants was 40.5 (standard deviation 14.7) years. Background data are presented in Table 1. Nearly 3 in 10 did not have any formal schooling. Although the occupation of men was diverse, almost 8 in 10 women were home makers. One in 5 men were from occupational categories of day-labourer (23.4%), business (20.3%), and cultivation (18.9%). One in 5 (21.3%) were overweight or obese (BMI > =25.0 kg/meter^2^).

**Table 1:** Social and other risk factors (number, percent) in adults, cross-sectional national survey in Bangladesh, 2015

Risk factors	Urban	Rural	Men	Women	All
	(***n*** = 716)	(***n*** = 1127)	(***n*** = 892)	(***n*** = 951)	(***n*** = 1843)
Age, years					
18–34	296 (41.3)	415 (36.8)	217 (24.3)	494 (51.9)	711 (38.6)
35–54	306 (42.7)	468 (41.5)	422 (47.3)	352 (37.0)	774 (42.0)
55–99	114 (15.9)	244 (21.7)	253 (28.4)	105 (11.0)	358 (19.4)
Occupation					
Homemakers	254 (35.5)	495 (43.9)	0 (0.0)	749 (78.8)	749 (40.7)
Laborer	81 (11.3)	161 (14.3)	209 (23.4)	33 (3.5)	242 (13.1)
Business professional	111 (15.5)	75 (6.7)	181 (20.3)	5 (0.5)	186 (10.1)
Service holder	96 (13.4)	29 (2.6)	98 (11.0)	27 (2.8)	125 (6.8)
Rickshaw/ Auto-Rick/ Van puller	27 (3.8)	44 (3.9)	70 (7.9)	1 (0.1)	71 (3.8)
Cultivator	5 (0.7)	164 (14.6)	168 (18.9)	1 (0.1)	169 (9.2)
Others	142 (19.8)	159 (14.1)	166 (18.6)	135 (14.2)	301 (16.3)
Education level					
No formal education (0)	143 (20.0)	421 (37.4)	267 (29.9)	297 (31.2)	564 (30.6)
Any primary education (1–5)	131 (18.3)	325 (28.8)	233 (26.1)	223 (23.4)	456 (24.7)
Any secondary education (6–10)	232 (32.4)	306 (27.2)	246 (27.6)	292 (30.7)	538 (29.2)
Above secondary (> = 11)	210 (29.3)	75 (6.7)	146 (16.4)	139 (14.6)	285 (15.5)
Wealth index quartiles^a^					
1st	75 (10.5)	401 (35.6)	206 (23.1)	270 (28.4)	476 (25.8)
2nd	108 (15.1)	354 (31.4)	220 (24.7)	220 (25.4)	462 (25.1)
3rd	203 (28.4)	245 (21.7)	231 (25.9)	217 (22.8)	448 (24.3)
4th	330 (46.1)	127 (11.3)	235 (26.3)	222 (23.3)	457 (24.8)
Overweight (body mass index≥25 Kg/m^2^)	225 (31.4)	167 (14.8)	148 (16.6)	244 (25.7)	392 (21.3)
History of physical trauma^b^	78 (10.9)	100 (8.9)	88 (9.9)	90 (9.5)	178 (9.7)
Smoking, ever	207 (28.9)	392 (34.9)	583 (65.4)	16 (1.7)	599 (32.5)
Diabetes mellitus^c^	73 (10.2)	36 (3.2)	50 (5.6)	59 (6.2)	109 (5.9)
Strenuous physical activity^d^	124 (17.3)	230 (20.4)	336 (37.7)	18 (1.9)	354 (19.2)

^a^The wealth index was constructed using principal component analysis out of a list of 20 household assets (See Methods section for details);

^b^Physical trauma during last 12 months that needed medical treatment with or without residual damage, e.g., injuries due to accidents while travelling by road, trauma during occupational works while working in farming lands or factories, physical assault, etc.;

^c^Diabetes was defined as random capillary glucose level > =11.1 or medication for diabetes;

^d^Fifth quintile of the MET-minutes distribution of work-related physical activity. Commutation and leisure time physical activities were not considered because these were negligible contributors (See Methods section for details)

### MSK conditions

There were 561 people with rheumatic conditions (225 men and 336 women). Prevalence of any MSK disorder was 30.4% with 95% confidence interval (CI) 28.3–32.5, which was higher in women (35.3%, 32.3–38.4) compared to men (25.2%, 22.4–28.1) (Table 2). Low back pain including lumbar spondylosis (18.6%) and knee osteoarthritis (7.3%), and soft tissue rheumatism (3.8%) were the commonest conditions in sexes combined. The prevalence of rheumatoid arthritis was 1.6%. Among all conditions, low back pain and rheumatoid arthritis differed significantly between men and women. The gender difference for soft tissue rheumatism showed a borderline significance, 95% CI being 1.5–3.6 for men and 3.6–6.3 for women. Others were not significantly different as indicated by overlapping 95% confidence intervals.

**Table 2:** Prevalence and 95% confidence interval (CI) of musculoskeletal conditions in adults, cross-sectional national survey in Bangladesh, 2015^a^

Rheumatic	Men (n = 892)			Women (n = 951)			Both Sexes (n = 1843)^a^		
Disorders	n	%	95% CI	n	%	95% CI	n	%	95% CI
Rheumatoid arthritis	6	**0.7**	**0.1–1.2**	23	**2.4**	**1.4–3.4**	29	1.6	1.1–2.3
Spondyloarthropathy	13	1.5	0.7–2.2	10	1.1	0.4–1.7	23	1.3	0.8–1.9
Knee osteoarthritis	60	6.7	5.1–8.4	74	7.8	6.1–9.5	134	7.3	6.2–8.6
Low back pain^b^	126	**14.1**	**12.1–16.7**	217	**22.8**	**20.3–25.6**	343	18.6	16.9–20.5
Cervical spondylosis	14	1.6	0.8–2.4	11	1.2	0.5–1.8	25	1.4	0.8–1.9
Soft tissue rheumatism^c^	23	2.6	1.5–3.6	47	4.9	3.6–6.3	70	3.8	3.0–4.8
Adhesive capsulitis of shoulder joint	10	1.1	0.4–1.8	16	1.7	0.9–2.5	26	1.4	1.0–2.1
Connective tissue disorder	1	0.1	0–0.3	5	0.5	0.1–1.0	6	0.3	0.2–0.7
Other noninflammatory^d^	31	3.5	2.3–4.7	31	3.3	2.1–4.4	62	3.4	2.6–4.3
Other inflammatory^e^	7	0.8	0.2–1.4	14	1.5	0.7–2.2	21	1.1	0.8–1.7
Any rheumatic disorder	225	**25.2**	**22.4–28.1**	336	**35.3**	**32.3–38.4**	561	30.4	28.3–32.5

Prevalence shown in bold face having non-overlapping CIs are significantly different (*P* < 0.05) between sexes;

^a^Multiple diagnosis in 192 patients. Therefore, the total of diseases exceeds 561;

^b^This category includes lumbar spondylosis also because accurate differentiation was not feasible in the field situation, investigation facility, etc.;

^c^This includes subacromial bursitis, epicondylitis, trochanteric bursitis, anserine bursitis, and fibromyalgia;

^d^Osteoarthritis of hip, osteoarthritis of hands, traumatic arthritis, traumatic fracture related condition, trauma of ligaments and soft issue, vague symptoms of myalgia and muscle spasm, vertebral spine related sciatica, vertebral scoliosis, ill-defined rheumatic syndromes;

^e^Monoarthritis, oligoarthritis, polyarthritis, gout, palindromic rheumatism, adult Still’s disease, vasculitis, primary Sjogren syndrome

**Fig. 2:**
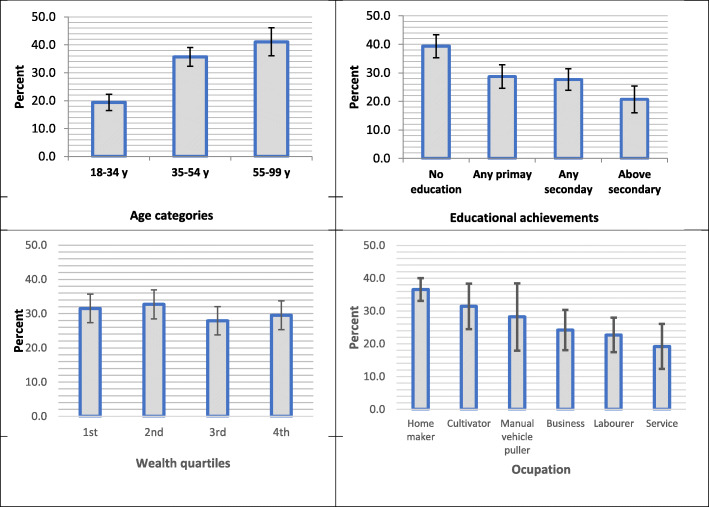
Prevalence of musculoskeletal conditions in adults according to age, educational achievement, economic status* and occupation, national cross-sectional survey in Bangladesh, 2015. *Wealth quartiles were created using household assets using principle component analysis

**Fig. 3:**
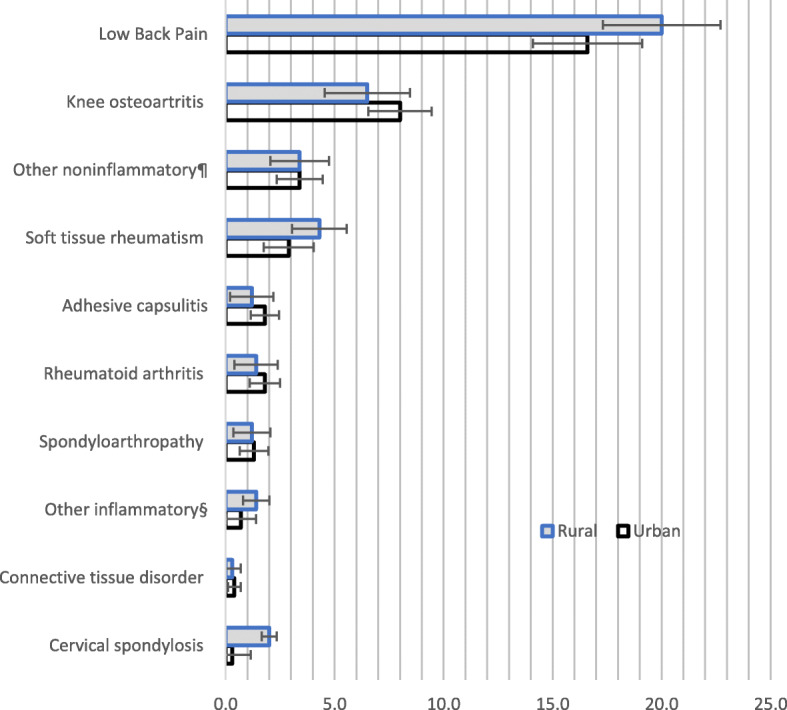
Prevalence of musculoskeletal conditions in adults according to urban-rural residence locations, cross-sectional national survey in Bangladesh, 2015. §includes osteoarthritis of hip, osteoarthritis of hand, traumatic arthritis, traumatic fracture related condition, trauma of ligament and soft issue, vague symptoms of myalgia and muscle spasm, vertebral spine related sciatica, vertebral scoliosis, ill-defined rheumatic syndromes; ¶includes monoarthritis, oligoarthritis, polyarthritis, gout, palindromic rheumatism

The prevalence of MSK in general increased with age, but it was inversely related to educational and economic status (Fig. 2). However, this relationship is very subtle in case of economic achievements. Certain occupations (such as home maker, cultivator and manual vehicle puller) had higher rates, but these differences were not statistically significant. Overall prevalence did not differ significantly between rural (31.1%, 28.4–33.8) and urban areas (29.5%, 26.1–32.8%). This was true for specific conditions also (Fig. 3)**.**

### Musculoskeletal pain

One-third men and women had musculoskeletal pain, although determined subjectively, and 9.1% of them had very severe pain. Low back (20.2%), knee (12.2%) and shoulder (6.2%) joints were the commonest site of pain. Next common sites were elbow (4.9%) and wrist (4.3%) joints (Table 3). We did not observe substantial difference in distribution of pain sites between rural and urban areas.

**Table 3:** Common musculoskeletal pain sites in adults, cross-sectional national survey in Bangladesh, 2015

Pain location	Urban (***n*** = 716)		Rural (***n*** = 1127)		Both (***n*** = 1843)	
	%	95% CI^**a**^	%	95% CI^**a**^	%	95% CI^**a**^
Whole body pain	1.0	0.3–1.7	1.4	0.7–2.1	1.2	0.7–1.7
Shoulder joint	5.0	3.4–6.6	6.9	5.4–8.4	6.2	5.1–7.3
Elbow joint	**2.5**	**1.4–3.6**	**6.5**	**5.1–7.9**	4.9	3.9–5.9
Wrist joint	3.1	1.8–4.4	5.1	3.8–6.4	4.3	3.4–5.2
Hand joints	3.4	2.1–4.7	4.3	3.1–5.5	4.0	3.1–4.9
Hip joint	0.7	0.1–1.3	0.8	0.3–1.3	0.8	0.4–1.2
Knee joint	10.6	8.3–12.9	13.2	11.2–15.2	12.2	10.7–13.7
Ankle joint	2.8	1.6–4.0	4.1	2.9–5.3	3.6	2.7–4.5
Foot joint	2.2	1.1–3.3	2.6	1.7–3.5	4.9	3.9–5.9
Neck	3.1	1.8–4.4	3.4	2.3–4.5	3.3	2.5–4.1
Upper back	4.3	2.8–5.8	3.2	2.2–4.2	3.6	2.7–4.5
Lower back	17.3	14.5–20.1	22.0	19.6–24.4	20.2	18.4–22
Chest	1.1	0.3–1.9	1.7	0.9–2.5	1.5	0.9–2.1
Arm	1.5	0.6–2.4	1.8	1.0–2.6	1.7	1.1–2.3
Forearm	1.0	0.3–1.7	2.5	1.6–3.4	1.9	1.3–2.5
Hand	1.4	0.5–2.3	2.0	1.2–2.8	1.8	1.2–2.4
Hip	0.7	0.1–1.3	0.3	0–0.6	0.4	0.1–0.7
Thigh	1.1	0.3–1.9	2.9	1.9–3.9	2.2	1.5–2.9
Leg	4.3	2.8–5.8	3.8	2.7–4.9	4.0	3.1–4.9
Foot	1.1	0.3–1.9	2.1	1.3–2.9	1.7	1.1–2.3

Percent figures shown in bold face having non-overlapping CIs are significantly different (*P* < 0.05) between urban-rural areas of residence;

^**a**^CI indicates confidence interval

### Disability and work loss

People with MSK conditions had a mean B-HAQ-DI score of 0.63. One-quarter (24.8%, 21.3–28.6) had some or much difficulty in doing their daily works defined by B-HAQ-DI score ≥ 0.8 (Table 4). None were in the ‘unable to do’ (B-HAQ score, 3.0) category. Their proportions were statistically similar between men (19.1%, 24.2–24.9) and women (28.6%, 23.9–33.8) with overlapping confidence intervals. The prevalence has shown an increasing trend with age, 14.9% in 18–34 years age group to 36.6% in 55+ year age group. Among the eight domains of B-HAQ-DI, the commonest problems were with daily works, walking and arising. Of those who had disability, 24.4% (21.0, 28.1) had work loss during last 12 months. Work loss in men (27.1%) and women (22.6%) was similar as indicated by overlapping confidence intervals. They had 12 median days of work loss during those 12 months.

**Table 4:** Disability and work loss among subjects with musculoskeletal conditions, cross-sectional national survey in Bangladesh, 2015^a^

Disability indices	Men (***n*** = 225)	Women (***n*** = 336)	Both, (***n*** = 561)
B-HAQ-DI^b^ score, mean	0.56 (0.47–0.65)	0.68 (0.61–0.76)	0.63 (0.56–0.71)
Disability (B-HAQ-DI Score ≥ 0.8)^c^, %	19.1 (14.2–24.9)	28.6 (23.9–33.8)	24.8 (21.3–28.6)
Any work loss (last 12 months), %	27.1 (21.7–33.3)	22.6 (18.5–27.4)	24.4 (21.0–28.1)
Duration of work loss (last 12 months)^d^, mean	14.7 (6.8–22.6)	9.7 (3.8–15.6)	11.7 (7.4–16.1)

^a^Results in the parentheses are 95% confidence intervals; None of the results are significantly different between sexes as indicated by overlapping confidence intervals;

^b^B-HAQ-DI indicates Bengali version of the Health Assessment Questionnaire Disability Index

^c^The cut-off point is according to Rosana Quintana (2016) [36]

^d^Number of days the incumbent had to stop working because of pain and related problems

### Factors associated with MSK conditions

Univariate logistic regression found significant relationship of six out of 11 candidate variables. These are age, education, smoking, strenuous physical activity, occupation, physical trauma, overweight and diabetes) as none of the 95% CIs of odds ratios included null values. However, in trivariate logistic regression analysis adjusted for age and sex, the significant relationship of education (odds ratio, 0.9, 0.8–0.9), overweight (1.5, 1.2–1.9), trauma (1.9, 1.4–2.6) and diabetes (1.5, 1.0–2.2) persisted (Table 5**)**. All other odds ratios became attenuated upon adjustment.

**Table 5:** Results of multiple logistic regression for musculoskeletal conditions combined, cross-sectional national survey in Bangladesh, 2015

Variables	Unadjusted		Age and sex adjusted	
	Odds ratio	95% confidence interval	Odds ratio	95% confidence interval
Age groups^a^	**1.73**	**1.51–1.99**		
Sex (women = 2/ men = 1)	**1.62**	**1.32–1.98**		
Education groups^b^	**0.76**	**0.69–0.83**	**0.87**	**0.78–0.96**
Wealth quartiles	0.95	0.87–1.04	0.98	0.90–1.08
Urban residence	1.08	0.88–1.32	1.00	0.81–1.24
Smoking, ever vs never	1.41	**1.13–1.75**	1.05	0.77–1.43
Strenuous physical activity^c^	**0.66**	**0.51–0.86**	0.97	0.71–1.32
Occupational groups^d^	**0.96**	**0.92–0.99**	1.00	0.95–1.05
Overweight^e^ (yes = 1/ no = 0)	**1.52**	**1.21–1.93**	**1.51**	**1.18–1.92**
History of trauma/ injury^f^ (yes = 1/ no = 0)	**1.81**	**1.32–2.49**	**1.88**	**1.36–2.62**
Diabetes^g^ (yes = 1/ no = 0)	**1.87**	**1.27–2.77**	1.47	0.98–2.20

Odds ratios with 95% confidence intervals that do not have null value (shown in bold face) are statistically significant (*P* < 0.05);

^a^18–34 y = coded as 1, 35–54 y = coded as 2, 55–99 y = coded as 3;

^b^No education = 1, any primary = 2, any secondary = 3, above higher secondary = 4

^c^Fifth quintile versus rest of MET minutes per week categories;

^d^See Table 1 for groups

^e^Body mass index≥25.0 kg/m^2^

^f^Physical trauma during last 12 months that needed medical treatment with or without residual damage, e.g., injuries due to accidents while travelling by road, trauma during occupational works while working in farming lands or factories, physical assault, etc

^g^Defined as random capillary glucose level > =11.1 or medication for diabetes

## Discussion

MSK conditions are among the most relevant health issues worldwide owing to the human suffering they impose, in addition to their increasing social and economic costs [50, 51]. In spite of the available evidence, MSK conditions are under-addressed in terms of programmatic approaches, treatment and health system’s response. We report here for the first time a nationally representative study in Bangladeshi adults addressing the equity issues related to age, sex and socio-economic status. We report here that three in ten Bangladeshi adults suffer from MSK conditions, and among them one in four has varying degrees of disability.

### Musculoskeletal pain

Pain was the commonest manifestation. Our survey questions were whether the respondents had pain, swelling and stiffness. Almost all 551 out of 561 had pain. Although remaning 10 subjects did not report pain, presence of pain could not be ruled out with certainty. They started taking NSAIDs or steroids advised by their doctors and continued taking these medicines overlapping our recall period of 7 days. Like our previous survey [23], we observed an increased frequency of pain with age. Women had higher frequency of pain compared to men, as was reported by most of the researchers. Similarly, the prevalence of musculoskeletal pain was greater in rural areas compared to urban areas. Commonest sites were low back (20.2%), knees and shoulders as in most other COPCORD studies including ours [23], except in India [21] and Iran [52]. There are many factors for high rates of low back pain. The most frequently reported factors are heavy physical workload such as lifting, awkward posture, lack of exercise and obesity [53] and age (especially above 35 years) [54]. Unidentified causes of high prevalence of low back pain in developing country may be vitamin D deficiency due to limited sun exposure and multiparity [55].

### *MSK* conditions

The most common MSK disorder in the study was low back pain (18.6%). The prevalence of low back pain was nearly like that of India [21], Kuwait [56] and Malaysia [57] but lower than in rural Iran [24] and urban Indonesia [15]. There are reports of higher prevalence of low back pain in occupations involving postural changes and weight-lifting [58–60]. The social culture of domestic and professional activities in bending posture may be responsible for higher prevalence of low back pain in Bangladesh but it needs further in-depth scrutiny. A rapid urbanization, transition to sedentary work and weight gain might also have contributed [61]. Contribution of related psychological factors like stress also remains to be studied in future [62].

Knee osteoarthritis (7.3%) was the second commonest rheumatic disorder. It may be related to more knee usage in our community during occupational and household chores, leisure and prayers [63]. Repetitive joint use and working in squatting position for prolonged time may be responsible for the high prevalence of knee osteoarthritis among homemakers, cultivators and manual vehicle (cycle rickshaw, cycle van, etc.) pullers. Climbing high stairs in urban areas might also be linked. This prevalence was lower than that of the urban Iran 15.3% [52] and higher than that of India (4.42%) [21] and Lebanon 3% [64]. Female gender, obesity and previous knee injury are recognized major risk factors of knee osteoarthritis. A study comparing risk factors among some of these countries might shed light on the cause of difference of the difference in the modifiable risk factors of knee osteoarthritis in the Asian countries.

Soft tissue rheumatism was in the third position in order of prevalence in our study. It had occupied the topmost position in COPCORD studies carried out among the Australian aborigines [65], Filipino [12] and Indonesian [13] rural studies. They have constituted major bulk of the MSK conditions in other Asia-Pacific COPCORD studies. Its high prevalence in the developing countries and in the rural communities may be explained by ergonomically inconvenient worker-workstation interface these settings [66]. In the present series, there was a big gender difference in the prevalence of soft tissue rheumatism. It may be explained by the inclusion of fibromyalgia (26 out of 70), a disease occurring exclusively in women, in this category of MSK conditions.

The prevalence of rheumatoid arthritis was 1.6%, women had significantly higher prevalence (2.4%) compared to men (0.7%). The prevalence reported in the previous Bangladesh COPCORD survey of 2005 was 0.9% [23]. Our current finding is close to that of Cuba (1.2%) [27] and Mexico (1.6%) [26]. The most contrasting findings have been reported from nearby Asian countries: India (0.5%) [21], Pakistan (0.6%) [18, 20], Thailand (0.1%) [19] and Malaysia (0.2%) [57]. The higher prevalence in our survey may be partly explained by the adoption of American College of Rheumatology/ European League Against Rheumatism (ACR/EULAR) 2010 classification criteria which has a higher sensitivity (97%) [34]. Prevalence of spondyloarthritis was 1.2%, which was also higher than in other COPCORD studies. Again, this might be attributed partly to the adoption of the new ASAS classification criteria which has a higher sensitivity (83%) [35].

### Factors related to rheumatic conditions

Education was a better discriminator than the wealth indices for MSK disorders in our sample. Educational achievement has been reported to have better rheumatoid arthritis outcome concerning pain and function [67]. Occupations demanding heavy physical work like homemaking, cultivation and rickshaw pulling had higher rates of complaints. Musculoskeletal problems were more common in subjects who performed heavy physical work and, particularly, in those in jobs that involve kneeling and squatting [68]. High BMI (≥ 25) was associated with higher musculoskeletal pain. Overweight and obese subjects had higher prevalence of pain in joints, knees, limbs and lower leg compared with normal weight subjects [69, 70]. History of trauma, as we observed, is associated with MSK conditions such as rheumatoid arthritis [71]. Diabetic patients had higher burden of musculoskeletal manifestations [72]. Diabetes affects the musculoskeletal system in multiple ways such as favoring hyperostosis, impacting joint mobility, neuropathy and microvascular diseases. Epidemiological studies support that MSK conditions are somehow associated with insulin resistance. It is well-known that many MSK conditions like adhesive capsulitis of shoulder, trigger finger, carpal tunnel syndrome etc. occur more frequently in diabetics compared to the non-diabetics. The association of diabetes with MSK conditions in our sample, however, disappeared in the age and sex-adjusted logistic model. It may be explained by the fact that our sample size was not adequate for multivariate regression modeling.

### Disability

In the current study the prevalence of functional disability was 24.8%. In the urban Iran (28.3%) the disability rate was close to this study [25]. The disability rates were a little lower in the earlier Bangladesh COPCORD survey (24%) [23]. Our rates in both the studies are much higher than rural Philippines (1.8%) [14] and rural Thailand (3%) [19]. These large differences may partly be explained by differences in definitions and methodologies used to detect functional disability [23], social custom, differences in occupation and workplace environment.

## Limitations

It was sometimes difficult to distinguish closely resembling conditions with the field epidemiological definitions. Most of the recognized classification criteria demand some investigations which were sometimes not possible due to lack of facilities in nearby locations and subjects’ unwillingness to travel long distance. For example, ASAS classification criteria for axial spondyloarthritis demand a few mandatory investigations [40], which were not possible in some cases. The sample size estimation for this survey was based on prevalence of MSK conditions combined. Therefore, caution has to be exerted in interpreting the results of individual conditions (specially the rare ones) especially when they are split in to four reporting domains.

Strengths: We used rheumatology residents as research physicians who had received extensive training before their deployment to the field. Most diagnoses made by them were double checked by the investigators. One visit of the investigators to each PSU was mandatory to validate their diagnosis and sort out confusing cases.

## Conclusions

Three in ten Bangladeshi adults suffer from MSK conditions. Low educational status, overweight and history of trauma are the factors to be targeted for interventions. This nationally representative survey warrants health system’s greater attention for addressing the challenges of pain and disabilities associated with MSK conditions. Further studies are needed to estimate the impact of this group of conditions particularly addressing related disabilities and loss of works.

## Data Availability

All data generated or analyzed during this study are included in this published article [and its supplementary information files].

## Abbreviations

ACR: American College of Rheumatology
ASAS: Assessment of SpondyloArthritis international Society
BMI: Body mass index
CASPAR: ClASsificationcriteria for Psoriatic ARthritis
CI: Confidence interval
COPCORD: Community Oriented Programme for Control of Rheumatic Disorders
DALY: Disability adjusted life years
EULAR: European League Against Rheumatism Collaborative Initiative
LMIC: Low- and middle-income countries
MET: Metabolic equivalent task
MSK: Musculoskeletal
OA: Osteoarthritis
OR: Odds ratio
PSU: Primary sampling unit
STEPS: Step-wise surveillance
WHO: World Health Organization
YLD: Years of life with disability
